# The evaluation of goal-directed activities to promote well-being and health in heart failure: EUROIA scale

**DOI:** 10.1186/s41687-024-00723-x

**Published:** 2024-04-29

**Authors:** Robert P. Nolan, Fatima Syed, Nicolette Stogios, Robert Maunder, Sanjeev Sockalingam, E. Shyong Tai, Mark Cobain, Rachel G. Peiris, Ella Huszti

**Affiliations:** 1 Behavioral Cardiology Research Unit, Peter Munk Cardiac Centre and Toronto General Hospital Research Institute, University Health Network, Toronto, ON Canada; 2 Institute of Medical Science, University of Toronto Faculty of Medicine, Toronto, ON Canada; 3 Department of Psychiatry, University of Toronto, Toronto, ON Canada; 4 Department of Psychiatry, Sinai Health System, Toronto, ON Canada; 5 Department of Education, Centre for Addiction and Mental Health, Toronto, ON Canada; 6 Division of Endocrinology, National University Hospital, Singapore, Singapore; 7 Younger Lives, London, UK; 8 Biostatistics Research Unit, University Health Network, Toronto, ON Canada

**Keywords:** Health-Related Quality-of-life, Goal-Directed activities, Well-being, Patient-reported outcome, Chronic heart failure

## Abstract

**Background:**

The **E**val**U**ation of goal-di**R**ected activities to pr**O**mote well-be**I**ng and he**A**lth (EUROIA) scale is a novel patient-reported measure that was administered to individuals with chronic heart failure (CHF). It assesses goal-directed activities that are self-reported as being personally meaningful and commonly utilized to optimize health-related quality of life (HRQL). Our aim was to evaluate psychometric properties of the EUROIA, and to determine if it accounted for novel variance in its association with clinical outcomes.

**Methods:**

This study was a secondary analysis of the CHF-CePPORT trial, which enrolled 231 CHF patients: median age = 59.5 years, 23% women. Baseline assessments included: EUROIA, Kansas City Cardiomyopathy Questionnaire–Overall Summary (KCCQ-OS), Patient Health Questionnaire–9 for depression (PHQ-9), and the Generalized Anxiety Disorder–7 (GAD-7). 12-month outcomes included health status (composite index of incident hospitalization or emergency department, ED, visit) and mental health (PHQ-9 and GAD-7).

**Results:**

Exploratory Principal Axis Factoring identified four EUROIA factors with satisfactory internal reliability: i.e., activities promoting eudaimonic well-being (McDondald’s ω = 0.79), social affiliation (⍺=0.69), self-affirmation (⍺=0.73), and fulfillment of social roles/responsibilities (Spearman-Brown coefficient = 0.66). Multivariable logistic regression indicated that not only was the EUROIA inversely associated with incidence of 12-month hospitalization/ED visits independent of the KCCQ-OS (Odds Ratio, OR = 0.95, 95% Confidence Interval, CI, 0.91, 0.98), but it was also associated with 12-month PHQ-9 (OR = 0.91, 95% CI, 0.86, 0.97), and GAD-7 (OR = 0.94, 95% CI, 0.90, 0.99) whereas the KCCQ-OS was not.

**Conclusion:**

The EUROIA provides a preliminary taxonomy of goal-directed activities that promote HRQL among CHF patients independently from a current gold standard state-based measure.

**Clinical trial registration:**

NCT01864369; https://classic.clinicaltrials.gov/ct2/show/NCT01864369.

**Supplementary Information:**

The online version contains supplementary material available at 10.1186/s41687-024-00723-x.

## Introduction

Health-related quality of life (HRQL) refers to an individual’s appraisal of the degree to which a pathophysiologic condition or treatment influences their physical and mental well-being. We previously observed that questionnaire items that make up HRQL assessments reflect an eclectic mix of features from foundational theories of well-being [1]. These include (i) hedonic elements: whether one’s experience has been positive (vs. negative) for mood, affect, and physical status, including an appraisal of being satisfied with life, (ii) eudaimonic components: the appraisal that one has been able to flourish in personal competencies, or thrive in social relationships, and (iii) markers of desire-satisfaction: whether subjective experience has been skewed towards satisfaction vs. frustration of desires.

### Mid-level theories of well-being and HRQL

Alexandrova’s philosophical work on the science of well-being [2] highlights how conventional assessments are commonly modeled on high-level, normative theories that do not extend beyond an abstract definition of well-being. That is, numerous psychometric assessments follow a convention of evaluating an individual’s self-reported state of well-being [3, 4] or HRQL [5, 6] according to the degree to which it deviates from idealized features of eudaimonism, hedonism, or a state of fulfilled desire. These profiles are abstract in the sense that content is independent of the situational context that characterizes an individual’s life, such as whether one is young or elderly, or financially secure or in poverty. Accordingly, the operative reference for well-being or HRQL in this approach is a state where (i) functional limitations are absent, (ii) positive emotions of joy, happiness, and life satisfaction are a normative experience while emotional distress, depressed mood, or physical pain are absent, (iii) there is an ability to flourish in being creative and productive without being burdened by personal struggle, (iv) social life is characterized by positive or loving relationships with a spouse, family, friends, and acquaintances at home and at work, and (v) a sense of purpose in life is sustained by a feeling of accomplishment and mastery over life’s challenges. In contrast, Alexandrova [2] has advocated the development of mid-level theories to address how well-being is understood or pursued by individuals in the situations that influence their daily life.

### Process- and state-based models of HRQL

Exploratory studies have provided some support for midlevel theories of how well-being is pursued. A series of studies in self-determination theory [7] examined individual aspirations for living well. Intrinsic aspirations were defined as life goals that were self-rewarding as valued ends in themselves, due to their ability to promote well-being by fulfilling basic psychological needs for autonomy, competence, or social connectedness. Extrinsic aspirations were those that were valued for their instrumental association with outcomes such as fame, wealth, or social influence, where the life goal was regulated by factors outside of an individual’s control. Over time, the pursuit of intrinsic aspirations was associated with numerous indicators of well-being such as increased positive affect, social affiliation, and perceived meaning in life [8, 9]. A similar research initiative on the pursuit of well-being in daily life was undertaken in a population survey of middle aged adults living in the United States [10]. Self-reported daily activities for living well were positively associated with physical health indices and health behaviors.

**Fig. 1:**
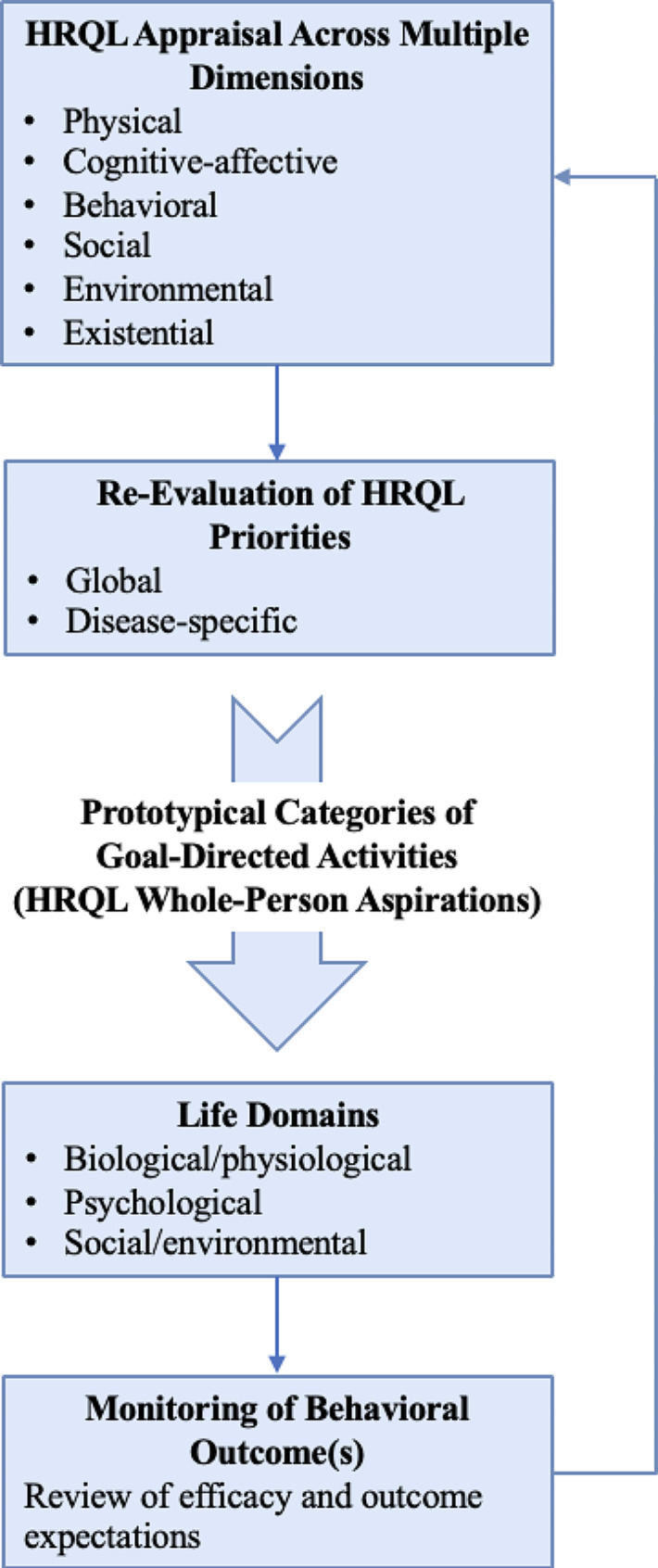
The process-based model of health-related quality of life (HRQL). (i) Appraisals of HRQL or well-being can be spontaneous or triggered by unexpected (“dynamic”) changes in health status or psychosocial demands [1]; (ii) these appraisals evoke heightened awareness of personally salient priorities for living well, which are associated with our pursuit of eudaimonic or hedonic well-being, the satisfaction of desires, or management of existential dread; (iii) these aspirations prompt us to initiate, adjust, or sustain goal-directed activities that aim to maintain or improve our well-being, and to date, a taxonomy of prototypical categories for these goal-directed activities is not established; (iv) each goal-directed activity is associated with perceived changes in bio-psycho-social domains of our environment; (v) these changes shape our efficacy and outcome expectations, which in turn influence re-appraisals of HRQL and ongoing efforts to self-regulate events that have the capacity to maintain or optimize our well-being

A recent theoretical paper by our team introduced a process-based model of HRQL [1]. It was based on a content analysis of current HRQL assessments, a philosophical study of HRQL and well-being, and a summary of qualitative research findings. The qualitative findings noted how patients described HRQL as an adaptive process where they pursued well-being via ongoing adjustments to dynamic (unanticipated) changes in their health status and associated life events. Their effort to *live well* was expressed as an ongoing iterative process of initiating, monitoring, evaluating, and revising goal-directed activities to maintain or improve their well-being. Figure 1 provides a meta-theoretical illustration of the self-regulatory process of living well, which includes (i) the appraisal of well-being in response to salient change in our biopsychosocial environment [11, 12], (ii) the initiation or adjustment of goal-directed activities to maintain or improve well-being within our specific life situation, in keeping with one’s salient life goals [8, 9], (iii) the evaluation of the effect of our goal-directed activities on our bio-psychosocial environment, which influences our outcome and efficacy expectations [7, 13], and (iv) subsequent re-appraisals of our well-being or HRQL which continues this self-regulatory cycle.

A state-based model of well-being has a notable influence in the fields of health policy [14], health economics [15], neuroscience [16] and psychology [17]. It defines well-being as a subjective experience that is comprised of features of eudaimonism, hedonism, and fulfilled desire. It also refers to “…how people experience and evaluate their lives and specific domains and activities in their lives” [18]. Ongoing research has aimed to identify distinct profiles of cognitive-emotional, neurophysiologic, and behavioral features that are hypothesized to comprise the state of subjective well-being [19, 20].

The current study re-focused the research agenda for well-being and HRQL, shifting it away from assessing an individual’s self-reported state of well-being, towards the goal of specifying a taxonomy of prototypical activities that are pursued to live well, as self-reported by individuals. Notably, these activities may be associated with the pursuit of a conventionally defined state of well-being as described above. In addition, activities for living well can be directed towards experiences that are extrinsic to notions of hedonic satisfaction or eudaimonic flourishing, as when one endures a stressful routine to support or care for a loved one, or when one responds to an adverse condition of life (e.g. discrimination, poverty, illness) by engaging in a routine that is personally or existentially meaningful while accepting its associated hardships. Our objective was to develop a descriptive assessment tool to evaluate a critical component of the process-based model. We examined goal-directed activities that are self-reported to promote HRQL and well-being among individuals diagnosed with chronic heart failure (CHF): the **E**val**U** ation of goal-diRected activities to prOmote well-beIng and heAlth (EUROIA).

## Methods

### Data source and study population

This investigation was a secondary analysis of a double-blind, digital health trial that aimed to improve self-care behavior and HRQL in CHF patients–the Canadian e-Platform to Promote Behavioral Self-Management in Chronic Heart Failure (CHF-CePPORT; see Appendix 1 for STROBE checklist) [21]. The sample estimate for this trial was based on the Heart Failure: A Controlled Trial Investigating Outcomes of Exercise Training (HF-ACTION) trial [22]. 231 patients were recruited from heart function clinics at hospitals in Toronto, Ottawa, and Vancouver, Canada, and enrolled based on diagnosis of CHF (ejection fraction ≤ 45% and New York Heart Association Class II or III). CHF-CePPORT [21] was approved by research ethics boards at each hospital. All participants provided informed consent. They were ≥ 18 years of age, medically stable for at least 1 month prior to enrolment, and fluent in English. Exclusion was based on severe comorbidities that would impede regular engagement with the digital intervention (e.g., acute renal failure, major psychiatric disorder, substance abuse). Patients on a heart transplant waitlist at the time of enrolment were also excluded.

### Assessments: baseline and 12-month

Participants were administered the following assessments at baseline: EUROIA, Kansas City Cardiomyopathy Questionnaire (KCCQ) [5], Physical Activity Scale for the Elderly (PASE) [23], 6-Minute Walk Test (6-MWT) [24], 7-Item Generalized Anxiety Disorder Questionnaire (GAD-7) [25], 9-Item Patient Health Questionnaire (PHQ-9) [26], Self-Care Behaviour Checklist for CHF of adherence to (i) planned exercise up to 150 min/wk, (ii) active living habits 5 to 6 d/wk, (iii) dietary intake of 3 to 5 vegetable servings/day, (iv) 2 to 4 fruit servings/day, and (v) < 30% calories from fat at each meal, (vi) omission of added salt to food at each meal, (vii) avoidance of salty snacks or fast foods, (viii) checking labels when shopping for food that is low in sodium and fat, and high in fiber, (ix) taking medications as prescribed, (x) recording weight each morning, (xi) limiting fluids to < 2 L (or 8 cups)/day, (xii) smoke-free lifestyle, and (xiii) ≤ 1 drink of alcohol/day [21]. At 12 months, we assessed health status using electronic patient records at participating hospitals: composite index of the incidence of HF hospitalization or all-cause emergency department (ED) visit. All-cause ED attendance was selected due to the co-morbid nature of CHF and the wide-ranging symptoms that are associated with this condition. We also assessed 12-month measures of mood (PHQ-9 [26]) and affect (GAD-7 [25]) as markers of mental health.

The EUROIA is a 13-item questionnaire comprised of goal-directed activities that promote HRQL and well-being. Individuals are asked to rate each goal-directed activity in terms of its frequency and importance/priority in their pursuit of living well. EUROIA items were selected based on qualitative research findings regarding common activity themes that are reported to promote HRQL, and key philosophical/theoretical themes of well-being that are embedded in conventional HRQL assessments: i.e. hedonic and eudaimonic well-being, desire satisfaction, and self-care activities that promote HRQL (Table 1)—see [1] for details. EUROIA items are gender-neutral and easily interpretable, with a Flesch Kincaid [27] reading level of 7.5. A 1-month timeframe was used for self-ratings of activity frequency using a Likert-type scale: 0 = not at all, 1 = 1 day per week, 2 = 2–3 days per week, and 3 = 4 or more days per week. Self-ratings of importance/priority were coded to provide a subjective valuation of the degree to which each activity in the EUROIA was meaningful or valued as contributing to their effort to live well: -1 = Not at all important, 0 = somewhat important, 1 = very important, and 2 = extremely important. The frequency * priority cross product provided a composite index where frequency ratings for each goal-directed activity for living well was weighted by the respondent’s subjective valuation. This coding procedure was modelled after previous psychometric instruments that used a weighting methodology for a given construct such as behaviors for valued living [28], daily stressful events [29], or health status [30]. Moreover, the self-ratings for items on the frequency * priority factors had a positive numeric value only if a given activity was appraised as contributing meaningfully to the respondent’s effort to live well.

**Table 1:** Self-rated responses for the EUROIA

1. EUROIA Items	EUROIA Self-Ratings		
	Frequency	Priority	Frequency * Priority
	Mdn (IQR)	Mdn (IQR)	Mdn (IQR)
1. …continue to grow and be productive in work or professional activities.	3.0 (0, 3)	1 (0, 2)	3.0 (0, 6)
2. …fulfill your role and responsibilities to support your family.	3.0 (0, 3)	2.0 (1, 2)	6.0 (3, 6)
3. …be physically active in your daily routine (e.g., walking, climbing stairs).	3.0 (3, 3)	2.0 (1, 2)	6.0 (3, 6)
4. …exercise a few times each week (e.g., brisk walking, swimming).	2.0 (1, 3)	1.0 (0, 2)	3.0 (0, 6)
5. …look and feel healthy and attractive.	3.0 (2, 3)	1.0 (1, 2)	3.0 (2, 6)
6. …do a special hobby or activity each week for personal satisfaction.	2.0 (1, 3)	1.0 (0, 2)	2.0 (0, 6)
7. …practice a skill to be excellent in an art, sport, craft, or learning activity.	2.0 (0, 3)	1.0 (0, 1)	0.5 (0, 3)
8. …enjoy pleasurable experiences of life (e.g., fine foods, art, or travel).	2.0 (1, 3)	1.0 (0, 2)	2.0 (0, 3.5)
9. …manage your emotions to be calm and relaxed as you went through each day.	3.0 (3, 3)	1.0 (1, 2)	3.0 (2, 6)
10. …participate in social activities to feel close to family or friends.	2.0 (1, 3)	1.0 (1, 2)	2.0 (1, 4)
11. …be of help to loved ones or those in need through your daily actions or words.	3.0 (2, 3)	1.0 (1, 2)	3.0 (2, 6)
12. …do an activity each week that connects you to a greater purpose in life.	1.5 (1, 3)	1.0 (0, 1)	1.0 (0, 3)

EUROIA = **E**val**U**ation of goal-di**R**ected activities to Pr**O**mote well-Be**I**ng and he**A**lth, Mdn = Median, IQR = Interquartile range (Q1, Q3)

Frequency ratings: 0 = Not at all, 1 = 1 time/week, 2 = 2–3 times/week, 3 = ≥ 4 times/week

Priority ratings: -1 = not at all important, 0 = somewhat important, 1 = very important, 2 = extremely important

Median (Interquartile Range) = Frequency, 3 (2, 4), Priority, 1 (1, 2), and Frequency*Priority, 3 (2, 4.5)

Psychometric assessments included in our study have been used extensively with cardiac populations. The 23-item KCCQ [5] assesses 6 domains of HRQL, of which we included Social Limitations (SL), Quality of Life (QL), Total symptoms (TS) and the Overall Summary (OS) subscales. The PHQ-9 [26] assesses symptoms of depression and the GAD-7 [25] measures anxiety, where higher scores indicate greater severity. The 6-MWT [24] is an objective measure of physical functioning. The PASE [23] measures functional capacity and leisure activities, where higher scores reflect greater physical functioning.

### Hypotheses

Our primary hypothesis for this exploratory study was that a summary index of the EUROIA would account for unique variance, independent of an established state-based HRQL assessment (the KCCQ [5]), when examining its association with a 12-month clinical outcome (Composite index of hospitalization or ED visit). Secondary hypotheses were that a summary index of the EUROIA would be associated with established indices of well-being or HRQL and decreased psychological distress, as measured by the KCCQ [5]), PHQ-9, GAD-7 [25], 6-MWT [24], PASE [23], and a Self-Care Behaviour Checklist [21]. We hypothesized that the magnitude of these associations would be in the low-to-moderate range, in keeping with similar behavioral research findings [31], and since the correlational data pertained to constructs that were theoretically distinct from the construct of goal-directed activities for living well that are measured by the EUROIA.

### Statistical analyses

All analyses were performed using IBM SPSS Statistics for Windows, Version 28.0. Statistical significance was defined as *p* < 0.05, 2-sided. The primary authors had full access to the data in the study and take responsibility for its integrity and data analysis. Clinical indices at baseline and 12 months were assessed for skewness and transformed, when necessary, into tertiles or clinically relevant categories. Descriptive statistics for interquartile range (IQR) are presented as (Q1, Q3). It was necessary to transform the PHQ-9 [26] and GAD-7 [25]: 0–4, asymptomatic vs. ≥ 5, mild or greater severity. The full CHF-CePPORT [21] sample was included in all analyses. Multiple imputation of missing data was not performed in the original trial because it would have invalidated planned analyses of the association between patient engagement (logon hours) with digital counseling and 12-month outcomes. The distribution of each item comprising the frequency (F), priority (P), and frequency*priority (F*P) EUROIA scales was evaluated in terms of median, and interquartile range.

Exploratory Principal Axis Factor (ePAF) analyses were conducted following Bartlett’s test of sphericity, to determine whether the EUROIA variables deviated significantly from an identity matrix, and after the Kaiser-Meyer-Olkin (KMO) test for the proportion of variance among the EUROIA items that was attributable to common variance. The underlying factor structure of the EUROIA was examined in separate ePAF’s for F, P, and F*P, and the factor solutions required eigenvalues > 1 and scree plot examination. Promax (oblique) rotation was used to allow for correlated factors, as uncorrelated factors are rare in health research [32]. The ePAF for the F*P scale provided an estimate of the frequency to which individuals engaged in goal-directed activities that were weighted by their self-rated importance/priority for living well. Accordingly, the F*P scale was the main index used in analyses of convergent validity, and of the association between the EUROIA with background characteristics and 12-month outcomes. Each factor score from the ePAF of the EUROIA F*P scale was obtained using regression scoring and then converted to T-scores for these analyses.

### EUROIA and background characteristics

The potential moderating influence of background characteristics on the EUROIA was examined using Pearson coefficients (r) for the correlation between the EUROIA F*P and normally distributed variables. Kendall’s tau (τ_b_) was used for skewed or categorical variables.

### Convergent validity of the EUROIA

Pearson coefficients for the EUROIA F*P summary score and F*P factors derived from the ePAF were assessed for the association with variables of interest: KCCQ subscales [5], PASE [23], 6-MWT [24], PHQ-9 [26], GAD-7 [25], and the CHF self-care checklist.

### Clinical outcomes at 12 months

Separate multivariable logistic regression analyses evaluated whether the EUORIA F*P summary score was independently associated with 12-month outcomes for health status (composite index of incident hospitalization/ED visit) and markers of mental health (GAD-7 [25] and PHQ-9 [26], which were categorized as asymptomatic, 0–4, vs. mild or greater symptom severity, ≥ 5). Each model included the baseline levels of the respective outcomes for GAD-7 [25] and PHQ-9 [26], as well as baseline LVEF (for the health status outcome), and baseline KCCQ-OS, age, and treatment arm.

The ePAF models yielded similar results with the Promax and Varimax rotation methods. Promax rotation was used in our final analyses since it accounted for correlated factors, and it was thought to be most appropriate and clear. Bias was controlled in this secondary analysis of the CHF-CePPORT trial. Research personnel had minimal contact with patients who were enrolled using a double-blind procedure, and the perception of support from our digital platform was similar for all subjects through automated frequency of email messages.

## Results

Details for background characteristics of the CHF-CePPORT sample were reported in the primary outcome paper [21]. Briefly, 231 were randomized to Usual Care (*n* = 114, 50.2%) or Digital Counseling (*n* = 117 49.8%), median age = 59.5 years (interquartile range, IQR: 52, 69), with *n* = 53 (23.0%) identifying as women, *n* = 162 (75.0%) reporting white ethno-racial background, *n* = 167 (72%) for post-secondary education, and gross annual family income ($CA) reported as low, < $70,000 (*n* = 78, 38%), moderate, $70,000 to $99,999 (*n* = 63, 31%), or high ≥ $100,000 (*n* = 64, 31%). Baseline LVEF was < 35 (*n* = 106, 46%), 35 to 40 (*n* = 70, 30%), and 41 to 45 (*n* = 55, 24%). Median KCCQ-OS [5] was 83.2 (IQR: 68, 93).

### EUROIA

Table 1 presents the mean (SD) self-ratings for the EUROIA factors.

#### Factor structure: frequency ratings

An ePAF was conducted on the frequency ratings for the 13-item EUROIA. This resulted in a 5-factor solution that was not acceptable. Two factors each had two items, and one of these was complex, with one item loading on two factors. Reliability coefficients were < 0.60 for four of the factors. Examination of EUROIA items led to the exclusion of Q12 from further analyses: “…how often have I been able to maintain a close emotional or sexual relationship with my spouse or partner?”

In the revised 12-item EUROIA, the KMO measure of common variance was 0.76, and Bartlett’s test of sphericity was significant (χ² = 393.7, df = 66, *p* < 0.001), which indicated that it was appropriate to proceed with the ePAF.

Table 2 presents a four-factor solution from the ePAF. Goal-directed activities reflected the pursuit of eudaimonic well-being (EUD-WB), self-affirmation (SLF-AFF), social affiliation (SOC-AFF), and fulfillment of social roles and responsibilities (SOC-RR). Factors for SOC-AFF and SOC-RR were comprised of only 2 items. This was a potential weakness in the factor structure. However, we retained this result given that both factors were theoretically distinct, and we deemed that they could be important for the final ePAF of the F*P scale.

**Table 2:** Pattern matrix loadings of a principal axis factor analysis of the EUROIA: Frequency items

EUROIA Items	Factors					
	Eudaimonic Well-being		Self-Affirmation		Social Affiliation	Social Roles/ Responsibilities
Practice a skill so that you can aim to be excellent in an art, sport, craft, or learning activity?	**0.80**	-0.12		-0.02		0.05
Do a special hobby or activity each week for personal satisfaction?	**0.77**	0.04		-0.04		-0.04
Do an activity each week that connects you to a greater purpose in life?	**0.47**	0.04		0.20		-0.07
Enjoy pleasurable experiences of life (e.g., fine foods, art, or travel)?	**0.45**	0.13		0.08		-0.03
Exercise a few times each week (e.g., brisk walking, swimming)?	0.13	**0.59**		-0.16		0.02
Look and feel healthy and attractive?	0.09	**0.55**		-0.04		0.11
Be physically active in your daily routine (e.g., walking, climbing stairs)?	-0.14	**0.54**		0.15		-0.09
Manage your emotions so you were calm and relaxed as you went through each day?	-0.08	**0.35**		0.27		0.03
Be of help to loved ones or those in need through your daily actions or words?	0.03	-0.06		**0.78**		-0.01
Participate in social activities where you can feel close to family or friends?	0.16	0.08		**0.42**		0.06
Fulfill your role and responsibilities to support your family?	-0.002	-0.03		0.05		**0.78**
Continue to grow and be productive in your work or professional activities?	-0.04	0.05		-0.03		**0.42**

EUROIA = **E**val**U**ation of goal-di**R**ected activities to Pr**O**mote well-Be**I**ng and he**A**lth

Factor correlations: 1:2 = 0.54, 1:3 = 0.47, 1:4 = 0.08, 2:3 = 0.47, 2:4 = 0.20, 3:4 = 0.09

#### Factor structure: priority ratings

The KMO value of 0.87, and Bartlett’s test of sphericity (χ² = 736.8, df = 66, *p* < 0.001) confirmed that it was appropriate to proceed with the ePAF. A two-factor solution was found (Table 3). The self-rated importance of activities in the first factor reflected intrinsic goals for well-being: e.g., activities that promoted personal satisfaction, skill mastery, connection to a greater purpose, or feelings of being calm and relaxed. We interpreted two items about social activities as being consistent with an intrinsic focus, as they reflected aspirations in keeping with altruism/compassion (Q11, “…be of help to loved ones or those in need”) or affiliation/intimacy (Q10, “…feel close to family or friends”). The second factor of priority ratings reflected social-interpersonal well-being: e.g., a striving to “…fulfill social roles and responsibilities to your family” (interpersonal valuation) or being able to “grow and be productive in work or professional activities” (social vitality/flourishing). Three activities that indicated a holistic theme of vitality had loadings on both the intrinsic and social-interpersonal dimensions: i.e., being able to “…look and feel healthy and attractive” (Q5), engage in physical activity (Q3) or exercise (Q4).

**Table 3:** Pattern matrix loadings of a principal axis factor analysis of the EUROIA: Priority/Importance items

EUROIA Items	Factors	
	1	2
Do a special hobby or activity each week for personal satisfaction?	**0.83**	-0.19
Do an activity each week that connects you to a greater purpose in life?	**0.74**	-0.22
Practice a skill so that you can aim to be excellent in an art, sport, craft, or learning activity?	**0.73**	0.01
Enjoy pleasurable experiences of life (e.g., fine foods, art, or travel)?	**0.73**	-0.07
Manage your emotions so you were calm and relaxed as you went through each day?	**0.61**	0.14
Participate in social activities where you can feel close to family or friends?	**0.54**	0.03
Look and feel healthy and attractive?	**0.51**	**0.36**
Be of help to loved ones or those in need through your daily actions or words?	**0.51**	0.20
Fulfill your role and responsibilities to support your family?	-0.16	**0.85**
Continue to grow and be productive in your work or professional activities?	-0.14	**0.64**
Be physically active in your daily routine (e.g., walking, climbing stairs)?	**0.36**	**0.38**
Exercise a few times each week (e.g., brisk walking, swimming)?	**0.32**	**0.32**

EUROIA = **E**val**U**ation of goal-di**R**ected activities to Pr**O**mote well-Be**I**ng and he**A**lth

#### Factor structure: F*P scale

We imposed a four-factor solution in keeping with the ePAF result for frequency ratings, as this solution was more interpretable and theoretically/clinically useful. Nevertheless, a two-factor solution was also obtained from the automated ePAF (Appendix 2), which is potentially a second-order factor.

The KMO value of 0.86, and Bartlett’s test of sphericity (χ² = 689.7, df = 66, *p* < 0.001) again confirmed that it was appropriate to proceed with the ePAF (Table 4). As expected, the factor structure was virtually unchanged from the solution observed for frequency ratings. Goal-directed activities once again reflected four themes: SLF-AFF, EUD-WB, SOC-RR, and SOC-AFF. Only SOC-RR was comprised of two items. Moreover, the ePAF presented a simple factor solution, with only one item (Q8, activities that evoked pleasurable or satisfaction) loading on both EUD-WB and SOC-AFF.

**Table 4:** Pattern matrix loadings of a principal axis factor analysis of the EUROIA: Frequency * Priority scores

EUROIA Items	Factors			
	Self-Affiliation	Eudaimonic Well-being	Social Roles/ Responsibilities	Social Affiliation
Be physically active in your daily routine (e.g., walking, climbing stairs)?	**0.98**	-0.21	0.01	-0.06
Exercise a few times each week (e.g., brisk walking, swimming)?	**0.56**	0.11	-0.09	-0.03
Look and feel healthy and attractive?	**0.56**	0.15	0.15	0.02
Manage your emotions so you were calm and relaxed as you went through each day?	**0.41**	0.15	0.06	0.13
Practice a skill so that you can aim to be excellent in an art, sport, craft, or learning activity?	-0.10	**0.91**	0.14	-0.13
Do a special hobby or activity each week for personal satisfaction?	0.29	**0.52**	-0.01	0.06
Do an activity each week that connects you to a greater purpose in life?	-0.08	**0.51**	-0.05	0.25
Fulfill your role and responsibilities to support your family?	-0.02	-0.06	**0.95**	0.09
Continue to grow and be productive in your work or professional activities?	0.03	0.18	**0.49**	-0.10
Participate in social activities where you can feel close to family or friends?	-0.08	0.06	-0.04	**0.75**
Be of help to loved ones or those in need through your daily actions or words?	0.03	-0.06	0.14	**0.70**
Enjoy pleasurable experiences of life (e.g., fine foods, art, or travel)?	0.14	**0.31**	-0.16	**0.39**

EUROIA = **E**val**U**ation of goal-di**R**ected activities to Pr**O**mote well-Be**I**ng and he**A**lth

#### EUROIA F*P scale: Psychometric summary

McDonald’s omega for the full EUROIA was 0.85, which is satisfactory. Reliability coefficients for the four factors were adequate: McDonald’s omega, SLF-AFF = 0.73, EUD-WB = 0.79, SOC-AFF = 0.69, and Spearman-Brown coefficient for the two-item SOC-RR = 0.66.

#### Association with background characteristics

The EUROIA F*P scale was not significantly associated with potential confounding variables from background characteristics—Table 5.

**Table 5:** Association between background characteristics and the EUROIA: Frequency*Priority Scale

Background Characteristics	EUROIA Self-Ratings^†^
	Frequency * Priority Cross-Product
	τ_b_
Gender*	-0.06
Age	-0.06
Racial-ethnic group: white vs. other	0.01
Living situation: single vs. spouse/partner	-0.09
Annual family income tertile	0.08
Education: ≤ Secondary, College/Undergraduate, Post-Graduate	-0.07
Left Ventricular Ejection Fraction	0.03

EUROIA = **E**val**U**ation of goal-di**R**ected activities to Pr**O**mote well-Be**I**ng and he**A**lth, M = Mean, SD = Standard Deviation, KCCQ-OS = Kansas City Cardiomyopathy Questionnaire-Overall Summary

Annual Family Income Tertiles = 1: <$70k (*n* = 78%, 38%), 2: $70k - $99,999 (*n* = 63, 30.7%), 3: ≥ $100k (*n* = 64, 31.2%)

* The available response choices for ‘Gender’ on the background questionnaire were listed as: man, woman, and other (with an accompanying textbox for specification). Participants exclusively selected the options of ‘man’ and ‘woman’, with no participants choosing ‘other’

^†^ All correlations failed to reach statistical significance at *p* < 0.05

#### Convergent validity

As expected, there was thematic congruence between the EUROIA F*P factors and established assessments of similar content (Table 6). The summary F*P EUROIA scale was positively associated with KCCQ subscales [5] and the CHF self-care behavior checklist, and it was inversely associated with the PHQ-9 [26] and GAD-7 [25]. These correlations were statistically significant but of small magnitude. The EUROIA EUD-WB factor was positively associated with the PASE [23], which measures one’s ability to engage in skills-focused activities. SOC-RR was associated with the KCCQ-SL scale [5], which reflects engagement in social activities. SOC-AFF was correlated with the PASE [23], which includes individual and social activities for leisure or exercise. The SLF-AFF factor included items for physical activity, exercise, and emotion regulation. It was positively associated with the PASE [23], 6-MWT [24], and KCCQ-TS [5], and inversely associated with the PHQ-9 [26] and GAD-7 [25].

**Table 6:** Correlations between health-related quality-of-life indices and the EUROIA Frequency*Priority Scale

	EUROIA Self-Ratings Frequency * Priority Cross-Products				
	Total	EUD-WB	SOC-RR	SLF-AFF	SOC-AFF
2. Background Characteristics	r	r	r	r	r
3. KCCQ-Overall Summary	**0.29** ^**‡**^	0.23^**†**^	0.19^*^	0.26^**‡**^	0.16^*^
KCCQ-Quality of Life	**0.23** ^**†**^	0.20^**†**^	0.12	0.17^*^	0.16^*^
KCCQ-Social Limitations	**0.24** ^**‡**^	0.18^*^	**0.20** ^**†**^	0.20^**†**^	0.14
KCCQ-Total Symptoms	**0.24** ^**‡**^	0.18^*^	0.14	**0.23** ^**‡**^	0.08
Physical Activity Scale for the Elderly: PASE	**0.37** ^**‡**^	**0.34** ^**‡**^	0.16^*^	**0.36** ^**‡**^	**0.27** ^**‡**^
6-Minute Walk Test: 6MWT	0.16^*^	0.15^*^	0.11	**0.22** ^**†**^	0.09
Anxiety: GAD-7	**-0.15** ^**†**^	-0.13^*^	0.03	**-0.16** ^**†**^	-0.13^*^
Depression: PHQ-9	**-0.16** ^**†**^	-0.13^*^	-0.01	**-0.16** ^**†**^	-0.12^*^
Self-Care Behavior Checklist	**0.19** ^**†**^	0.14	-0.02	**0.31** ^**‡**^	0.07

^*^p < 0.05, ^†^*p* < 0.01, ^‡^*p* < 0.001

EUROIA = **E**val**U**ation of goal-di**R**ected activities to Pr**O**mote well-Be**I**ng and he**A**lth, M = Mean, SD = Standard Deviation, KCCQ-OS = Kansas City Cardiomyopathy Questionnaire, GAD-7 = Generalized Anxiety Disorder – 7 item scale, PHQ-9 = Patient Health Questionnaire – 9 item scale

#### Clinical outcomes at 12 months

Multivariable logistic regression models indicated that the EUROIA F*P summary score accounted for independent variance in its inverse association with 12-month outcomes. Baseline assessments for the EUROIA and KCCQ-OS [5] were associated with decreased risk (OR = 0.88, 95% confidence interval, CI, 0.80, 0.95; and OR = 0.95, 95% CI, 0.91, 0.98, respectively) for incidence of hospitalization or ED visit (Table 7). However, the EUROIA, but not the KCCQ-OS [5], had a significant inverse association with elevated symptoms (≥ mild vs. asymptomatic) at 12 months for depression (OR = 0.91, 95% CI, 0.86, 0.97) and anxiety (OR = 0.94, 95% CI, 0.90, 0.99) in the baseline adjusted models (Table 7).

**Table 7:** Multivariable Logistic Regression models for the association between 12-Month clinical outcomes and the EUROIA Frequency*Priority Scale

Variables	Odds Ratio	95% CI	*p*-value
	**Composite index of hospitalization or ED visit at 12-months**		
Baseline KCCQ-OS	0.95	0.91, 0.98	0.002
Baseline F*P EUROIA	0.88	0.80, 0.95	**0.002**
**Treatment Arms: DC vs. UC**	1.04	0.30, 3.55	0.96
**Age**	0.96	0.91, 1.00	0.09
**Baseline LVEF**	0.93	0.88, 1.00	**0.04**
	**PHQ-9 – Depression at 12 months**		
**Baseline KCCQ-OS**	1.01	0.98, 1.05	0.47
Baseline F*P EUROIA	0.91	0.86, 0.97	**0.002**
**Treatment Arms: DC vs. UC**	1.11	0.41, 3.01	0.83
**Age**	0.97	0.94, 1.01	0.20
**Baseline PHQ-9**	26.48	7.66, 93.72	**< 0.001**
	**GAD-7 – Anxiety at 12 months**		
**Baseline KCCQ-OS**	0.99	0.96, 1.02	0.41
Baseline F*P EUROIA	0.94	0.90, 0.99	**0.02**
**Treatment Arms: DC vs. UC**	1.14	0.45, 2.92	0.78
**Age**	0.96	0.93, 0.995	**0.02**
**Baseline GAD-7**	8.09	3.12, 20.99	**< 0.001**

KCCQ-OS = Kansas City Cardiomyopathy Questionnaire – Overall Summary; EUROIA = **E**val**U**ation of goal-di**R**ected activities to pr**O**mote well-be**I**ng and he**A**lth; Treatment Arms: Usual Care (UC) = 0 (*N* = 73), Digital Counseling (DC) = 1 (*N* = 73); ED = Emergency department; LVEF = Left Ventricular Ejection Fraction

PHQ-9 = Patient Health Questionnaire for depression-9 item: 0 = minimal symptoms (< 5), 1 = mild or greater (≥ 5); GAD-7 = General Anxiety Disorder Questionnaire-7 item: 0 = minimal symptoms (< 5), 1 = mild or greater (≥ 5)

#### Goal-directed activities for HRQL and “frequency-priority fit”

The median value for F*P self-ratings for the 12 EUROIA activities was 0.54 (Interquartile Range, IQR, 0.36, 0.75). Similarly, the sum number of EUROIA activities that were self-rated as being “very important” or “extremely important” in the pursuit of living well had a median of 4 (IQR, 1, 7) out of 12 potential activities. These metrics (approximately, 4 to 6 activities) provide a preliminary index of frequency-priority fit for goal-directed activities for living well that promote HRQL, as defined by markers of improved health and mental health status, as noted above.

## Discussion

This study provides empirical support for a new psychometric instrument, the EUROIA, that presents a taxonomy of goal-directed activities associated with the pursuit of well-being (living well). These activities are an essential component of a process-based model of HRQL (Fig. 1). Among a sample of individuals diagnosed with CHF, the EUROIA demonstrated a simple factor structure based on ePAF for F, P, and F*P scales, and there was evidence of satisfactory convergent validity and internal reliability for the F*P scale. In addition, the EUROIA was associated with 12-month markers of physical and mental health, as defined by reduced risk for incident HF hospitalization or all-cause ED visit, and for elevated symptoms of depression (PHQ-9) [26] or anxiety (GAD-7) [25]. Importantly, the EUROIA accounted for variance in these 12-month measures that was independent of variance attributable to an established gold standard for HRQL assessment – the KCCQ-OS [5].

The process-based framework of the EUROIA introduces a new research agenda aimed at evaluating how an individual is pursuing HRQL and well-being in their daily life. Importantly, this approach open up new opportunities for patient-centered feedback. For example, the EUROIA F*P profile could be used to assist individuals in making an informed decision about whether to maintain their current repertoire of goal-directed activities for living well, or to explore additional categories of activities to improve or sustain their HRQL. It also has the potential to be used prescriptively. It may be possible to incorporate feedback from the EUROIA in cognitive-behavioral procedures such as behavioral activation, when prescribing activities with patients that aim to enhance their HRQL, or to improve their mood and affect [33].

Three specific findings of this study bear further discussion. First, the EUROIA was developed from a conceptual framework that differs from conventional HRQL assessments. In usual practice, individuals self-rate their functional status across bio-psycho-social domains, and this profile is evaluated in reference to an idealized HRQL state—i.e. complete understanding of how to manage a medical condition, absence of any limitation in mobility or social and sexual functioning, and absence of emotional distress or dissatisfaction with life–c.f. KCCQ [5]. In contrast, the EUROIA exemplifies a process-based approach to health and well-being (Fig. 1) which is grounded in a lineage of clinical research that produced evidence-based models of one’s ongoing effort to maintain or improve health and well-being through adaptive, self-determined activities within a complex system of reciprocal person-environment interactions. These models highlight how an individual’s appraisal of HRQL reflects an iterative process where we initiate, monitor, evaluate, and maintain or revise our activities to promote well-being, by means of: (i) self-managing illness-related stress and utilizing our perceived resources for living well [11, 12], (ii) fulfilling our basic psychological needs for autonomy, relatedness, and competence [7], or (iii) optimizing our perceived efficacy in managing person-environment interactions [13].

Second, the EUROIA includes self-ratings of the frequency (F) to which an individual engages in goal-directed activities for living well. Individuals also self-rate each activity according to its perceived priority/importance (P) in their overall pursuit of this goal (living well). To our knowledge, the EUROIA is distinct from other HRQL assessments in so far as it includes weighted frequency scores in the F*P index for each goal-directed activity. In effect, these F*P ratings identify activities that are personally salient and meaningful to the individual’s pursuit of increased HRQL or well-being—which is arguably an essential point of reference when planning or evaluating patient-centered care.

We interpreted the association between the EUROIA F*P scale and baseline HRQL (KCCQ-OS) [5] or 12-month markers of physical and mental health (hospitalization/ED visit, PHQ-9 and GAD-7), as being due to the individual’s pursuit of goal-directed activities that are “self-concordant” with their intrinsic values [34]. Confirmatory findings on this point have been reported from cross-cultural research where it was shown that life goals that reflect an individual’s intrinsic values are associated with improved health and well-being [35]. Our present results add to the view that there is therapeutic benefit of having self-concordant life goals. In addition, the EUROIA F*P scale is distinct from previous research on aspirations/life goals in so far as it identifies activities that serve as mid-level processes through which our aspirations are pursued in daily life–c.f. Alexandrova [2].

Third, ePAF results for the EUORIA found four principal themes of goal-directed activity, which can be interpreted as a preliminary taxonomy for goal-directed activities that are pursued to maintain or improve HRQL or well-being. The initial three themes are extensively discussed in HRQL research, therefore comments on them will be succinct.

### Eudaimonic well-being

It is noteworthy for a process-based approach to HRQL that Aristotle introduced the concept of *eudaimonia* as an activity (vs. an idealized state) that was expressed through individual flourishing and living in a manner that exemplified character virtues of justice, courage, moderation, and wisdom [1]. This concept was introduced to modern research on well-being by Ryff [36] and later by Ryan and Deci [37], but the concept of personal flourishing also has roots in humanistic theories such as Maslow’s description of a self-actualizing life [38], or Rogers’ notion of actualizing oneself to “become (our) potentialities” [39]. The therapeutic value of eudaimonic activities in promoting health and well-being has been well-documented within the positive psychology movement [37, 40].

### Social affiliation

Social affiliation was defined in the EUROIA by activities that promote social attachment or intimacy with significant others. Recent research suggests that affiliation evokes the perception of being valued by others, which contributes to the perception of having a meaningful life and having increased access to instrumental support. Activities that promote social affiliation or support are a major feature of active emotion-focused coping, which facilitates improved HRQL [41], and a decreased risk for depression and anxiety [42] as well as mortality [43].

### Fulfillment of social roles and responsibilities

The SOC-RR factor in the EUROIA included activities through which individuals seek to fulfill perceived responsibilities or roles that are meaningful in their interactions within their family network or workplace. Prilleltensky and colleagues [44] have reported that our perception of social worth and individual well-being is motivated not only by the need to be valued by others, but by the need to contribute something of value to the common good in the context of our interpersonal, familial, communal, or societal relationships. This theory grows out of a philosophical perspective developed by Sen [45] which highlights how our capacity to engage in activities that enable individuals to flourish is moderated by constraints and opportunities in our socio-economic environment. Accordingly, activities reflected in the SOC-RR theme have been reported to serve as a protective mechanism against emotional distress and decreased HRQL [46, 47].

### Self-affirmation

In considering the EUROIA SELF-AFF factor, it is necessary to recall that our sample was comprised of individuals diagnosed with a chronic progressive medical condition, CHF, which is characterized by successive episodes (often unanticipated) of acute symptoms or medical crises that may require hospitalization, and these events are followed by periods of stability that become progressively shortened over time [48]. Qualitative research findings illustrate how it is common for persons with CHF to feel that life is tenuous due to regular reminders of their existential threat, and this in turn contributes to psychological distress as well as the motivation to recover a sense of purpose and meaning in their new life, post-diagnosis [1].

We suggest that activities aimed at maintaining or improving HRQL for our cohort of CHF patients have profound significance as part of their effort to recover a sense of personal agency and to assert their autonomy. EUROIA items in the SELF-AFF category included engaging in exercise or physical activity, doing something to feel healthy and attractive, or managing emotions to promote a sense of calm or relaxation. This dimension of the EUROIA has particular relevance for the pursuit of well-being among individuals who are contending with adversity or suffering from causes that include nonmedical sources.

Limitations for this study include the need to demonstrate the generalizability of the current findings beyond CHF patients, to diverse populations, in terms of medical condition, ethno-racial groups, age, and gender. The current version of the EUROIA identified four clinical/theoretical categories of goal-directed activities for living well. Follow-up research is needed to build upon the present findings with the EUROIA in order to fully explore and establish its validity, reliability, and clinical utility. Certainly, a confirmatory factor analysis will be required to establish a common metric for further assessing different model fits and to ensure that the proposed factor structures of the EUROIA are robust and replicable. It is possible that additional prototypical categories will emerge as new items are added that are tailored to the characteristics of cohorts with different background characteristics. It is also necessary to administer the EUROIA to a large sample in order to establish its construct validity, sensitivity to change, and clinical utility.

## Conclusion

Findings of this study support the convergent validity, internal reliability, and potential clinical utility of the EUROIA. It’s focus on goal-directed activities that are self-reported to promote well-being represents an important feature of a novel process-based model of HRQL. The EUROIA also introduced a preliminary taxonomy of prototypical categories of these goal-directed activities. There is clear potential to use the EUROIA as both a descriptive and prescriptive tool in applied HRQL research or in clinical settings.

### Electronic supplementary material

Below is the link to the electronic supplementary material.

Supplementary Material 1

Supplementary Material 2

## Data Availability

The data underlying this article cannot be shared publicly due to privacy and confidentiality guidelines that protect patients who participated in this study. Data will be available from the corresponding author pending approval of Research Ethics Boards of participating institutions and on reasonable request received from qualified researchers trained in human subject confidentiality protocols.

## Abbreviations

HRQL: Health-Related Quality of Life
CHF: Chronic Heart Failure
EUROIA: EvalUation of Goal-DiRected Activities to PrOmote Well-BeIng and HeAlth
CHF-CePPORT: Canadian e-Platform to Promote Behavioral Self-Management in Chronic Heart Failure
HF-ACTION: Heart Failure: A Controlled Trial Investigating Outcomes of Exercise Training
KCCQ: Kansas City Cardiomyopathy Questionnaire
PASE: Physical Activity Scale for the Elderly
6-MWT: The 6-Minute Walk Test
GAD-7: 7-Item Generalized Anxiety Disorder Questionnaire
PHQ-9: 9-Item Patient Health Questionnaire
ED: Emergency Department
KCCQ-SL: Kansas City Cardiomyopathy Questionnaire-Social Limitations
KCCQ-QL: Kansas City Cardiomyopathy Questionnaire-Quality of Life
KCCQ-TS: Kansas City Cardiomyopathy Questionnaire-Total Symptoms
KCCQ-OS: Kansas City Cardiomyopathy Questionnaire-Overall Summary
F: EUROIA Frequency Ratings
P: EUROIA Priority Ratings
F*P: EUROIA Frequency*Priority Cross-Product Ratings
ePAF: Exploratory Principal Axis Factoring Analysis
KMO: Kaiser-Meyer-Olkin Test
EUD-WB: Eudaimonic Well-being Factor
SLF-AFFIRM: Self-Affirmation Factor
SOC-AFFIL: Social Affirmation Factor
SOC-RR: Social Roles and Responsibilities Factor
