# Supplementary material for: The evaluation of goal-directed activities to promote well-being and health in heart failure: EUROIA scale
Source: J Patient Rep Outcomes. 2024 Apr 29;8:47. doi: 10.1186/s41687-024-00723-x (PMC11058156; doi:10.1186/s41687-024-00723-x)
Supplement: Supplementary file 1 — Supplementary Material 1 [file 41687_2024_723_MOESM1_ESM.docx]

**APPENDIX**

**Appendix: 2-Factor Pattern matrix loadings of a principal axis factor analysis of the EUROIA: Frequency * Priority scores.**

| **EUROIA Items** | **Factors** | |
| --- | --- | --- |
|  | **1** | **2** |
| Do a special hobby or activity each week for personal satisfaction? | **0.79** | -0.02 |
| Do an activity each week that connects you to a greater purpose in life? | **0.77** | -0.13 |
| Practice a skill so that you can aim to be excellent in an art, sport, craft, or learning activity? | **0.69** | -0.17 |
| Enjoy pleasurable experiences of life (e.g., fine foods, art, or travel)? | **0.69** | -0.10 |
| Manage your emotions so you were calm and relaxed as you went through each day? | **0.57** | 0.06 |
| Participate in social activities where you can feel close to family or friends? | **0.50** | 0.21 |
| Look and feel healthy and attractive? | **0.48** | **0.36** |
| Be of help to loved ones or those in need through your daily actions or words? | **0.47** | 0.09 |
| Fulfill your role and responsibilities to support your family? | **0.44** | **0.30** |
| Continue to grow and be productive in your work or professional activities? | -0.23 | **0.96** |
| Be physically active in your daily routine (e.g., walking, climbing stairs)? | -0.02 | **0.49** |
| Exercise a few times each week (e.g., brisk walking, swimming)? | **0.36** | **0.38** |

EUROIA = **E**val**U**ation of goal-di**R**ected activities to Pr**O**mote well-Be**I**ng and health
