# Supplementary material for: The evaluation of goal-directed activities to promote well-being and health in heart failure: EUROIA scale
Source: J Patient Rep Outcomes. 2024 Apr 29;8:47. doi: 10.1186/s41687-024-00723-x (PMC11058156; doi:10.1186/s41687-024-00723-x)
Supplement: Supplementary file 2 — Supplementary Material 2 [file 41687_2024_723_MOESM2_ESM.docx]

STROBE Statement—checklist of items that should be included in reports of observational studies

|  | **Item No.** | **Recommendation** | **Page  No.** | **Relevant text from manuscript** |
| --- | --- | --- | --- | --- |
| **Title and abstract** | 1 | (*a*) Indicate the study’s design with a commonly used term in the title or the abstract | 1 | “This study was a secondary analysis of the CHF-CePPORT trial.” |
|  |  | (*b*) Provide in the abstract an informative and balanced summary of what was done and what was found | 1 | **“Background:** The EvalUation of goal-diRected activities to prOmote well-beIng and heAlth (EUROIA) scale is a novel patient-reported measure that was administered to individuals with chronic heart failure (CHF). It assesses goal-directed activities that are self-reported as being personally meaningful and commonly utilized to optimize health-related quality of life (HRQL). Our aim was to evaluate psychometric properties of the EUROIA, and to determine if it accounted for novel variance in its association with clinical outcomes.  **Methods:** This study was a secondary analysis of the CHF-CePPORT trial, which enrolled 231 CHF patients: median age = 59.5 years, 23% women. Baseline assessments included: EUROIA, Kansas City Cardiomyopathy Questionnaire–Overall Summary (KCCQ-OS), Patient Health Questionnaire–9 for depression (PHQ-9), and the Generalized Anxiety Disorder–7 (GAD-7). 12-month outcomes included health status (composite index of incident hospitalization or emergency department, ED, visit) and mental health (PHQ-9 and GAD-7).  **Results:** Exploratory Principal Axis Factoring identified four EUROIA factors with satisfactory internal reliability: i.e., activities promoting eudaimonic well-being (McDondald’s ω=0.79), social affiliation (⍺=0.69), self-affirmation (⍺=0.73), and fulfillment of social roles/responsibilities (Spearman-Brown coefficient=0.66). Multivariable logistic regression indicated that not only was the EUROIA inversely associated with incidence of 12-month hospitalization/ED visits independent of the KCCQ-OS (Odds Ratio, OR = 0.95, 95% Confidence Interval, CI, 0.91, 0.98), but it was also associated with 12-month PHQ-9 (OR = 0.91, 95% CI, 0.86, 0.97), and GAD-7 (OR = 0.94, 95% CI, 0.90, 0.99) whereas the KCCQ-OS was not.  **Conclusion:** The EUROIA provides a preliminary taxonomy of goal-directed activities that promote HRQL among CHF patients independently from a current gold standard state-based measure.” |
| **Introduction** | | | |  |
| Background/rationale | 2 | Explain the scientific background and rationale for the investigation being reported | 6  7 | “A recent theoretical paper by our team introduced a process-based model of HRQL. It was based on a content analysis of current HRQL assessments, a philosophical study of HRQL and well-being, and a summary of qualitative research findings. The qualitative findings noted how patients described HRQL as an adaptive process where they pursued well-being via ongoing adjustments to dynamic (unanticipated) changes in their health status and associated life events. Their effort to *live well* was expressed as an ongoing iterative process of initiating, monitoring, evaluating, and revising goal-directed activities to maintain or improve their well-being. Figure 1 provides a meta-theoretical illustration of the self-regulatory process of living well, which includes (i) the appraisal of well-being in response to salient change in our biopsychosocial environment, (ii) the initiation or adjustment of goal-directed activities to maintain or improve well-being within our specific life situation, in keeping with one’s salient life goals [8; 9], (iii) the evaluation of the effect of our goal-directed activities on our bio-psychosocial environment, which influences our outcome and efficacy expectations, and (iv) subsequent re-appraisals of our well-being or HRQL which continues this self-regulatory cycle.”  “The current study re-focused the research agenda for well-being and HRQL, shifting it away from assessing an individual’s self-reported state of well-being, towards the goal of specifying a taxonomy of prototypical activities that are pursued to live well, as self-reported by individuals. Notably, these activities may be associated with the pursuit of a conventionally defined state of well-being as described above.” |
| Objectives | 3 | State specific objectives, including any prespecified hypotheses | 8  10 | “Our objective was to develop a descriptive assessment tool to evaluate a critical component of the process-based model. We examined goal-directed activities that are self-reported to promote HRQL and well-being among individuals diagnosed with chronic heart failure (CHF): the **E**val**U**ation of goal-di**R**ected activities to pr**O**mote well-be**I**ng and he**A**lth (EUROIA).”  “Our primary hypothesis for this exploratory study was that a summary index of the EUROIA would account for unique variance, independent of an established state-based HRQL assessment (the KCCQ), when examining its association with a 12-month clinical outcome (Composite index of hospitalization or ED visit). Secondary hypotheses were that a summary index of the EUROIA would be associated with established indices of well-being or HRQL and decreased psychological distress, as measured by the KCCQ), PHQ-9, GAD-7, 6-MWT, PASE, and a Self-Care Behaviour Checklist. We hypothesized that the magnitude of these associations would be in the low-to-moderate range, in keeping with similar behavioral research findings, and since the correlational data pertained to constructs that were theoretically distinct from the construct of goal-directed activities for living well that are measured by the EUROIA.” |
| **Methods** | | | |  |
| Study design | 4 | Present key elements of study design early in the paper | 8 | “This investigation was a secondary analysis of a double-blind, digital health trial that aimed to improve self-care behavior and HRQL in CHF patients--the Canadian e-Platform to Promote Behavioral Self-Management in Chronic Heart Failure.” |
| Setting | 5 | Describe the setting, locations, and relevant dates, including periods of recruitment, exposure, follow-up, and data collection | 8 | “231 patients were recruited from heart function clinics at hospitals in Toronto, Ottawa, and Vancouver, Canada.”  “Assessments: Baseline and 12-month.”  Details regarding periods of recruitment are in the original CHF-CePPORT trial as follows: “Recruitment began in January 2014. Written informed consent from patients was obtained during enrollment. Final 12-month assessments were completed in February 2018.” |
| Participants | 6 | (*a*) *Cohort study*—Give the eligibility criteria, and the sources and methods of selection of participants. Describe methods of follow-up  *Case-control study*—Give the eligibility criteria, and the sources and methods of case ascertainment and control selection. Give the rationale for the choice of cases and controls  *Cross-sectional study*—Give the eligibility criteria, and the sources and methods of selection of participants | 8 | “The sample estimate for this trial was based on the Heart Failure: A Controlled Trial Investigating Outcomes of Exercise Training (HF-ACTION) trial [22]. 231 patients were recruited from heart function clinics at hospitals in Toronto, Ottawa, and Vancouver, Canada, and enrolled based on diagnosis of CHF (ejection fraction ≤ 45% and New York Heart Association Class II or III). CHF-CePPORT was approved by research ethics boards at each hospital. All participants provided informed consent. They were ≥ 18 years of age, medically stable for at least 1 month prior to enrolment, and fluent in English. Exclusion was based on severe comorbidities that would impede regular engagement with the digital intervention (e.g., acute renal failure, major psychiatric disorder, substance abuse). Patients on a heart transplant waitlist at the time of enrolment were also excluded.” |
|  |  | (*b*) *Cohort study*—For matched studies, give matching criteria and number of exposed and unexposed  *Case-control study*—For matched studies, give matching criteria and the number of controls per case |  | N/A |
| Variables | 7 | Clearly define all outcomes, exposures, predictors, potential confounders, and effect modifiers. Give diagnostic criteria, if applicable | 9  11 | “Participants were administered the following assessments at baseline: EUROIA, Kansas City Cardiomyopathy Questionnaire (KCCQ), Physical Activity Scale for the Elderly (PASE), 6-Minute Walk Test (6-MWT) [24], 7-Item Generalized Anxiety Disorder Questionnaire (GAD-7), 9-Item Patient Health Questionnaire (PHQ-9), Self-Care Behaviour Checklist for CHF of adherence to (i) planned exercise up to 150 min/wk, (ii) active living habits 5 to 6 d/wk, (iii) dietary intake of 3 to 5 vegetable servings/day, (iv) 2 to 4 fruit servings/day, and (v) <30% calories from fat at each meal, (vi) omission of added salt to food at each meal, (vii) avoidance of salty snacks or fast foods, (viii) checking labels when shopping for food that is low in sodium and fat, and high in fiber, (ix) taking medications as prescribed, (x) recording weight each morning, (xi) limiting fluids to <2 L (or 8 cups)/day, (xii) smoke-free lifestyle, and (xiii) ≤1 drink of alcohol/day. At 12 months, we assessed health status using electronic patient records at participating hospitals: composite index of the incidence of HF hospitalization or all-cause emergency department (ED) visit. All-cause ED attendance was selected due to the co-morbid nature of CHF and the wide-ranging symptoms that are associated with this condition. We also assessed 12-month measures of mood (PHQ-9) and affect (GAD-7) as markers of mental health.  The EUROIA is a 13-item questionnaire comprised of goal-directed activities that promote HRQL and well-being. Individuals are asked to rate each goal-directed activity in terms of its frequency and importance/priority in their pursuit of living well.”  “Clinical indices at baseline and 12 months were assessed for skewness and transformed, when necessary, into tertiles or clinically relevant categories. Descriptive statistics for interquartile range (IQR) are presented as (Q1, Q3). It was necessary to transform the PHQ-9 and GAD-7: 0-4, asymptomatic vs. ≥ 5, mild or greater severity. The full CHF-CePPORT sample was included in all analyses.” |
| Data sources/ measurement | 8* | For each variable of interest, give sources of data and details of methods of assessment (measurement). Describe comparability of assessment methods if there is more than one group | 10 | “Psychometric assessments included in our study have been used extensively with cardiac populations. The 23-item KCCQ assesses 6 domains of HRQL, of which we included Social Limitations (SL), Quality of Life (QL), Total symptoms (TS) and the Overall Summary (OS) subscales. The PHQ-9 assesses symptoms of depression and the GAD-7 measures anxiety, where higher scores indicate greater severity. The 6-MWT is an objective measure of physical functioning. The PASE measures functional capacity and leisure activities, where higher scores reflect greater physical functioning.” |
| Bias | 9 | Describe any efforts to address potential sources of bias | 13  10  12 | “Bias was controlled in this secondary analysis of the CHF-CePPORT trial. Research personnel had minimal contact with patients who were enrolled using a double-blind procedure, and the perception of support from our digital platform was similar for all subjects through automated frequency of email messages.”  “Psychometric assessments included in our study have been used extensively with cardiac populations.”  “The potential moderating influence of background characteristics on the EUROIA was examined using Pearson coefficients (r) for the correlation between the EUROIA F*P scale and normally distributed variables. Kendall’s tau (τ_b_) was used for analyses with skewed or categorical variables.” |
| Study size | 10 | Explain how the study size was arrived at | 8 | “The sample estimate for this trial was based on the Heart Failure: A Controlled Trial Investigating Outcomes of Exercise Training (HF-ACTION) trial.” |

Continued on next page

| Quantitative variables | 11 | Explain how quantitative variables were handled in the analyses. If applicable, describe which groupings were chosen and why | 11 | “Clinical indices at baseline and 12 months were assessed for skewness and transformed, when necessary, into tertiles or clinically relevant categories. Descriptive statistics for interquartile range (IQR) are presented as (Q1, Q3). It was necessary to transform the PHQ-9 and GAD-7: 0-4, asymptomatic vs. ≥ 5, mild or greater severity” |
| --- | --- | --- | --- | --- |
| Statistical methods | 12 | (*a*) Describe all statistical methods, including those used to control for confounding | 12-13 | “Exploratory Principal Axis Factor (ePAF) analyses were conducted following Bartlett’s test of sphericity, to determine whether the EUROIA variables deviated significantly from an identity matrix, and after the Kaiser-Meyer-Olkin (KMO) test for the proportion of variance among the EUROIA items that was attributable to common variance. The underlying factor structure of the EUROIA was examined in separate ePAF’s for F, P, and F*P, and the factor solutions required eigenvalues > 1 and scree plot examination. Promax (oblique) rotation was used to allow for correlated factors, as uncorrelated factors are rare in health research. The ePAF for the F*P scale provided an estimate of the frequency to which individuals engaged in goal-directed activities that were weighted by their self-rated importance/priority for living well. Accordingly, the F*P scale was the main index used in analyses of convergent validity, and of the association between the EUROIA with background characteristics and 12-month outcomes. Each factor score from the ePAF of the EUROIA F*P scale was obtained using regression scoring and then converted to T-scores for these analyses.  **EUROIA and background characteristics.** The potential moderating influence of background characteristics on the EUROIA was examined using Pearson coefficients (r) for the correlation between the EUROIA F*P and normally distributed variables. Kendall’s tau (τ_b_) was used for skewed or categorical variables.  **Convergent validity of the EUROIA.** Pearson coefficients for the EUROIA F*P summary score and F*P factors derived from the ePAF were assessed for the association with variables of interest: KCCQ subscales, PASE, 6-MWT, PHQ-9, GAD-7, and the CHF self-care checklist.  **Clinical outcomes at 12 months.** Separate multivariable logistic regression analyses evaluated whether the EUORIA F*P summary score was independently associated with 12-month outcomes for health status (composite index of incident hospitalization/ED visit) and markers of mental health (GAD-7 and PHQ-9, which were categorized as asymptomatic, 0 – 4, vs. mild or greater symptom severity, ≥ 5). Each model included the baseline levels of the respective outcomes for GAD-7 and PHQ-9, as well as baseline LVEF (for the health status outcome), and baseline KCCQ-OS, age, and treatment arm.” |
|  |  | (*b*) Describe any methods used to examine subgroups and interactions | N/A |  |
|  |  | © Explain how missing data were addressed | 11 | “Multiple imputation of missing data was not performed in the original trial because it would have invalidated planned analyses of the association between patient engagement (logon hours) with digital counseling and 12-month outcomes.” |
|  |  | (*d*) *Cohort study*—If applicable, explain how loss to follow-up was addressed  *Case-control study*—If applicable, explain how matching of cases and controls was addressed  *Cross-sectional study*—If applicable, describe analytical methods taking account of sampling strategy | N/A | The original CHF-CePPORT trial involved online assessments and automated email messages for 12 months. Participation was entirely in each aspect was voluntary. All data collected up to the point of loss to follow-up was utilized in the analyses. |
|  |  | (*e*) Describe any sensitivity analyses | 34 | Sensitivity analyses were conducted in the context of demonstrating convergent validity in Table 6 for the EUROIA questionnaire.  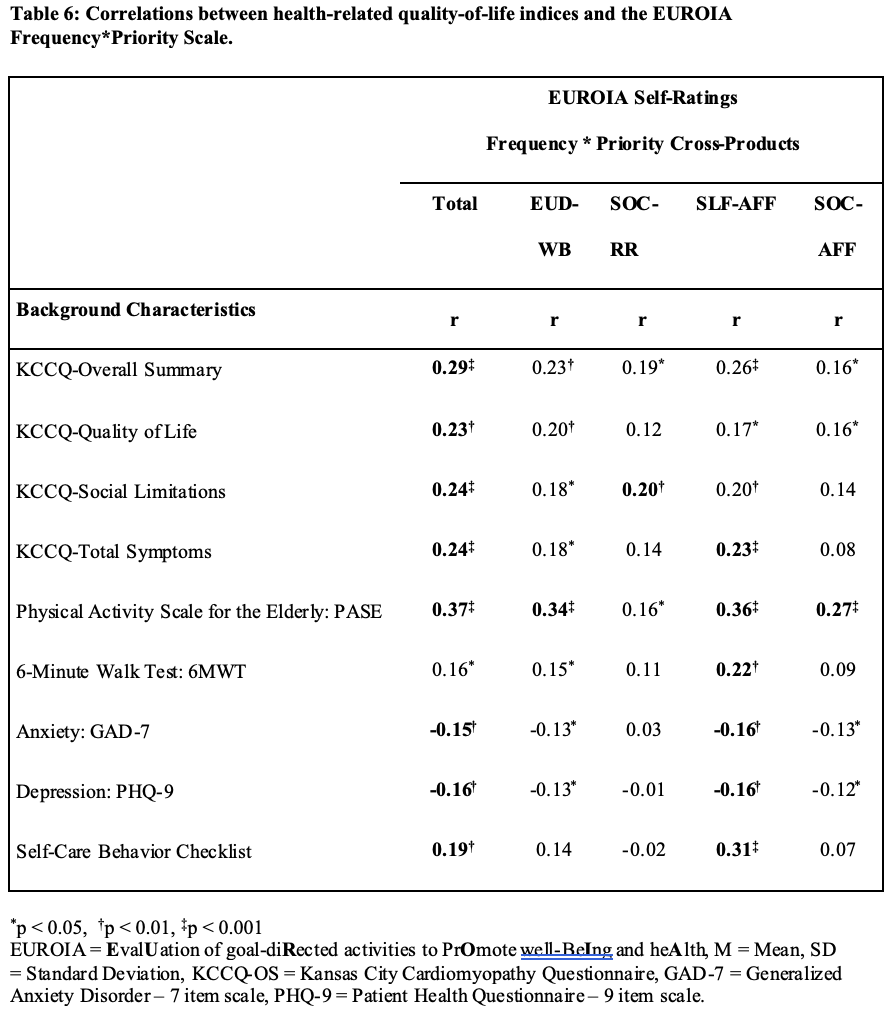 |
| **Results** | | | | |
| Participants | 13* | (a) Report numbers of individuals at each stage of study—eg numbers potentially eligible, examined for eligibility, confirmed eligible, included in the study, completing follow-up, and analysed | N/A | These details were elaborated upon are in the original CHF-CePPORT trial and are as follows: 3066 individuals were screened, and 1005 potential participants approached. 242 participants consented to participate and 122 were randomized to the control group and 120 to the intervention group. From the control group, 98 participants completed the 12-month assessment. From the intervention group, 100 participants completed the 12-month assessment. |
|  |  | (b) Give reasons for non-participation at each stage | N/A | These details were elaborated upon are in the original CHF-CePPORT trial and are as follows: Out of the 3066 individuals screened, 2061 failed to meet initial screening criteria. Out of the 1005 potential participants approached, 763 were excluded as they were ineligible (n = 345), declined to participate (n = 316), withdrew consent (n = 59), lost communication (n = 37), or had multiple comorbidities or LVEF >45% (n = 6). From the control group, 4 patients passed away during the trial and 4 received a heart transplant, making them ineligible, and 16 were lost to attrition. From the intervention group, 3 patients received heart transplants, making them ineligible, and 17 were lost to attrition. |
|  |  | (c) Consider use of a flow diagram | N/A | We are unable to utilize the flow diagram from the CHF-CePPORT trial in the manuscript as it was already published in the original trial article. However, we are adding the flow chart to the checklist for your review.  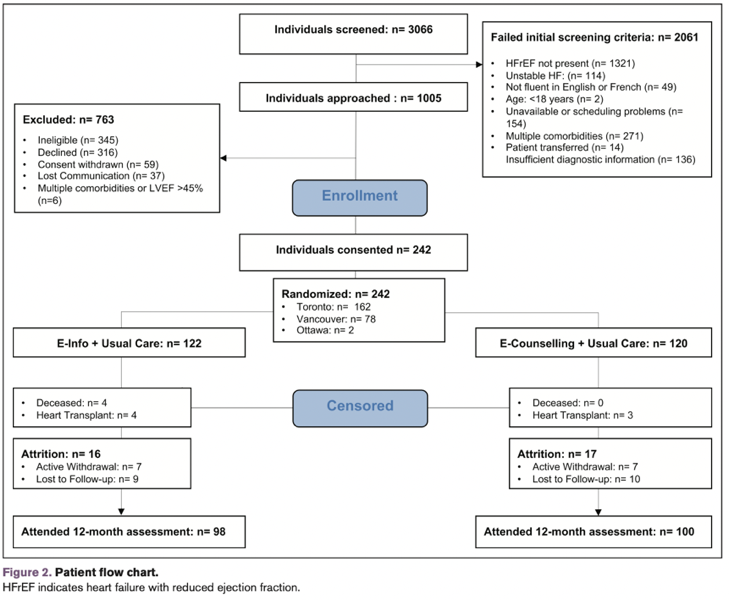 |
| Descriptive data | 14* | (a) Give characteristics of study participants (eg demographic, clinical, social) and information on exposures and potential confounders | 13 | “Details for background characteristics of the CHF-CePPORT sample were reported in the primary outcome paper. Briefly, 231 were randomized to Usual Care (n=114, 50.2%) or Digital Counseling (n=117 49.8%), median age = 59.5 years (interquartile range, IQR: 52, 69), with n=53 (23.0%) identifying as women, n=162 (75.0%) reporting white ethno-racial background, n=167 (72%) for post-secondary education, and gross annual family income ($CA) reported as low, < $70,000 (n=78, 38%), moderate, $70,000 to $99,999 (n=63, 31%), or high ≥ $100,000 (n=64, 31%). Baseline LVEF was < 35 (n=106, 46%), 35 to 40 (n=70, 30%), and 41 to 45 (n=55, 24%). Median KCCQ-OS [5] was 83.2 (IQR: 68, 93).” |
|  |  | (b) Indicate number of participants with missing data for each variable of interest |  | Missing data per variable of interest:  Baseline Scores:  EUROIA Scale : n = 49  KCCQ-OS: n = 1  GAD-7: n = 0  PHQ-9: n = 1  12-month indices:  Composite index of hospitalization and ED visits: n = 0  GAD-7: n = 5  PHQ-9: n = 3 |
|  |  | (c) *Cohort study*—Summarise follow-up time (eg, average and total amount) | N/A | Details provided in the original CHF-CePPORT trial are as follows: “Assessments were administered online at baseline, 4, and 12 months… and [patients] contacted by our program via email on a weekly basis for months 1 to 4, then bi-weekly for months 5 to 8, and monthly for months 9 to 12.” |
| Outcome data | 15* | *Cohort study*—Report numbers of outcome events or summary measures over time | N/A | Our outcome measures include the following: 12-month composite index of incident hospitalization and/or ED visit, and 12-month index of anxiety (GAD-7) or depression (PHQ-9). The trial was designed with assessments at baseline, 4-month, and 12-month, where 4-month outcomes were not included in this paper. |
|  |  | *Case-control study—*Report numbers in each exposure category, or summary measures of exposure |  |  |
|  |  | *Cross-sectional study—*Report numbers of outcome events or summary measures |  |  |
| Main results | 16 | (*a*) Give unadjusted estimates and, if applicable, confounder-adjusted estimates and their precision (eg, 95% confidence interval). Make clear which confounders were adjusted for and why they were included | 35 | 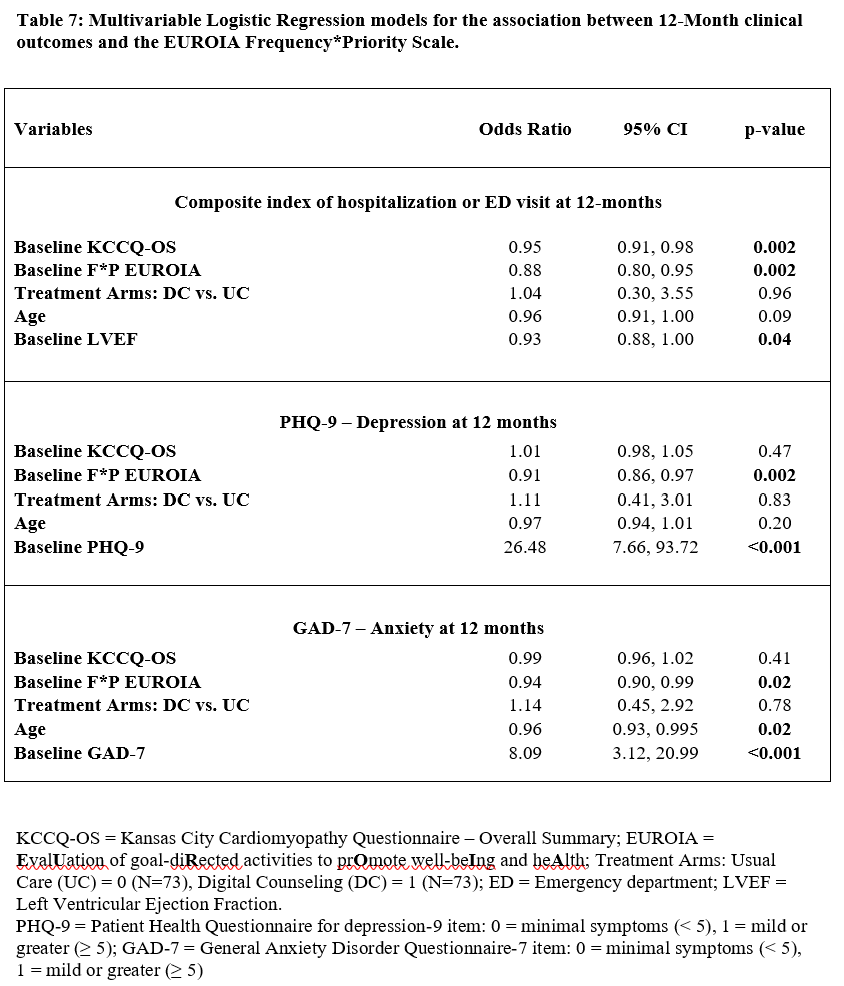 |
|  |  | (*b*) Report category boundaries when continuous variables were categorized | N/A |  |
|  |  | (*c*) If relevant, consider translating estimates of relative risk into absolute risk for a meaningful time period | N/A |  |

| Other analyses | 17 | Report other analyses done—eg analyses of subgroups and interactions, and sensitivity analyses | N/A |  |
| --- | --- | --- | --- | --- |
| **Discussion** | | | | |
| Key results | 18 | Summarise key results with reference to study objectives | 17 | “This study provides empirical support for a new psychometric instrument, the EUROIA, that presents a taxonomy of goal-directed activities associated with the pursuit of well-being (living well). These activities are an essential component of a process-based model of HRQL (Figure 1). Among a sample of individuals diagnosed with CHF, the EUROIA demonstrated a simple factor structure based on ePAF for F, P, and F*P scales, and there was evidence of satisfactory convergent validity and internal reliability for the F*P scale. In addition, the EUROIA was associated with 12-month markers of physical and mental health, as defined by reduced risk for incident HF hospitalization or all-cause ED visit, and for elevated symptoms of depression (PHQ-9) or anxiety (GAD-7). Importantly, the EUROIA accounted for variance in these 12-month measures that was independent of variance attributable to an established gold standard for HRQL assessment – the KCCQ-OS.” |
| Limitations | 19 | Discuss limitations of the study, taking into account sources of potential bias or imprecision. Discuss both direction and magnitude of any potential bias | 22 | “Limitations for this study include the need to demonstrate the generalizability of the current findings in more diverse populations, in terms of medical condition, ethno-racial groups, age, and gender. The current version of the EUROIA identified four clinical/theoretical categories of goal-directed activities for living well. Follow-up research is needed to build upon the present findings with the EUROIA in order to fully explore and establish its validity, reliability, and clinical utility. Certainly, a confirmatory factor analysis will be required to establish a common metric for further assessing different model fits and to ensure that the proposed factor structures of the EUROIA are robust and replicable. It is possible that additional prototypical categories will emerge as new items are added that are tailored to the characteristics of cohorts with different background characteristics. It is also necessary to administer the EUROIA to a large sample in order to establish its construct validity, sensitivity to change, and clinical utility.” |
| Interpretation | 20 | Give a cautious overall interpretation of results considering objectives, limitations, multiplicity of analyses, results from similar studies, and other relevant evidence | 18-20 | “The process-based framework of the EUROIA introduces a new research agenda aimed at evaluating how an individual is pursuing HRQL and well-being in their daily life. Importantly, this approach open up new opportunities for patient-centered feedback. For example, the EUROIA F*P profile could be used to assist individuals in making an informed decision about whether to maintain their current repertoire of goal-directed activities for living well, or to explore additional categories of activities to improve or sustain their HRQL. It also has the potential to be used prescriptively. It may be possible to incorporate feedback from the EUROIA in cognitive-behavioral procedures such as behavioral activation, when prescribing activities with patients that aim to enhance their HRQL, or to improve their mood and affect.  Three specific findings of this study bear further discussion. First, the EUROIA was developed from a conceptual framework that differs from conventional HRQL assessments. In usual practice, individuals self-rate their functional status across bio-psycho-social domains, and this profile is evaluated in reference to an idealized HRQL state—i.e. complete understanding of how to manage a medical condition, absence of any limitation in mobility or social and sexual functioning, and absence of emotional distress or dissatisfaction with life–c.f. KCCQ. In contrast, the EUROIA exemplifies a process-based approach to health and well-being (Figure 1) which is grounded in a lineage of clinical research that produced evidence-based models of one’s ongoing effort to maintain or improve health and well-being through adaptive, self-determined activities within a complex system of reciprocal person-environment interactions. These models highlight how an individual’s appraisal of HRQL reflects an iterative process where we initiate, monitor, evaluate, and maintain or revise our activities to promote well-being, by means of: (i) self-managing illness-related stress and utilizing our perceived resources for living well, (ii) fulfilling our basic psychological needs for autonomy, relatedness, and competence, or (iii) optimizing our perceived efficacy in managing person-environment interactions.  Second, the EUROIA includes self-ratings of the frequency (F) to which an individual engages in goal-directed activities for living well. Individuals also self-rate each activity according to its perceived priority/importance (P) in their overall pursuit of this goal (living well). To our knowledge, the EUROIA is distinct from other HRQL assessments in so far as it includes weighted frequency scores in the F*P index for each goal-directed activity. In effect, these F*P ratings identify activities that are personally salient and meaningful to the individual’s pursuit of increased HRQL or well-being—which is arguably an essential point of reference when planning or evaluating patient-centered care.  We interpreted the association between the EUROIA F*P scale and baseline HRQL (KCCQ-OS) or 12-month markers of physical and mental health (hospitalization/ED visit, PHQ-9 and GAD-7), as being due to the individual’s pursuit of goal-directed activities that are “self-concordant” with their intrinsic values. Confirmatory findings on this point have been reported from cross-cultural research where it was shown that life goals that reflect an individual’s intrinsic values are associated with improved health and well-being. Our present results add to the view that there is therapeutic benefit of having self-concordant life goals. In addition, the EUROIA F*P scale is distinct from previous research on aspirations/life goals in so far as it identifies activities that serve as mid-level processes through which our aspirations are pursued in daily life--c.f. Alexandrova.  Third, ePAF results for the EUORIA found four principal themes of goal-directed activity, which can be interpreted as a preliminary taxonomy for goal-directed activities that are pursued to maintain or improve HRQL or well-being. The initial three themes are extensively discussed in HRQL research, therefore comments on them will be succinct.” |
| Generalisability | 21 | Discuss the generalisability (external validity) of the study results | 22 | “Limitations for this study include the need to demonstrate the generalizability of the current findings beyond CHF patients…” |
| **Other information** | |  | | |
| Funding | 22 | Give the source of funding and the role of the funders for the present study and, if applicable, for the original study on which the present article is based | Included in the Title Page | “The work was supported by the Canadian Institutes of Health Research, Grant no. MOP-287717.” |

*Give information separately for cases and controls in case-control studies and, if applicable, for exposed and unexposed groups in cohort and cross-sectional studies.

**Note:** An Explanation and Elaboration article discusses each checklist item and gives methodological background and published examples of transparent reporting. The STROBE checklist is best used in conjunction with this article (freely available on the Web sites of PLoS Medicine at http://www.plosmedicine.org/, Annals of Internal Medicine at http://www.annals.org/, and Epidemiology at http://www.epidem.com/). Information on the STROBE Initiative is available at [www.strobe-statement.org](http://www.strobe-statement.org).
